# The American Dental Association

**Published:** 1891-05

**Authors:** J. N. Crouse

**Affiliations:** Chairman Ex. Com.


### THE AMERICAN DENTAL ASSOCIATION.

CHICAGO, May 25th, 1891.

*Editor of American Journal of Dental Science:*

A resolution was adopted at the last meeting of the American Dental Association, authorizing the Executive Committee to communicate with members of dental societies and with the dental profession of the country, to the end that the membership of the various local and State societies may be increased; that the usefulness of the American Dental Association may be advanced, and the relations between it and the local societies be made more intimate. One object is to awaken a lively interest in the meetings of the American Dental Association, and to secure further representation from the various local societies at the annual general meetings. Will you be kind enough to show your pride in your local society by interesting yourself personally in this matter, and secure the appointment of delegates to the next meeting of the American Dental Association, to be held at Saratoga Springs, N. Y., August 4th, 1891? In addition to the appointment of delegates we would suggest that your society appoint a committee who will forward to the chairman of each section such matters of interest as have transpired in your society during the year. The facts need only to be given to the chairman or secretary in such shape as to enable him to include them in the general report of each section. The names and addresses of the officers of the sections are herewith enclosed. If the societies of which you are a member have already met, will you send the paper you have read, or an abstract of it, or a brief outline of what you said in the course of discussion, or a short report of the entire meeting to some one of the chairmen or secretaries?

We desire a full attendance at the next meeting, as matters of great importance will come before it. Arrangements will be made to secure reduced railroad rates, but in order to get any reduction for return trip, it is *absolutely necessary to get a "certificate plan receipt" for full fare paid* at time of starting, of the agent of whom ticket is purchased. The best hotel rates will be secured, and all who visit Saratoga will, we hope, be well repaid for the time and money spent in visiting that famous resort.

Yours truly,

J. N. CROUSE, *Chairman Ex. Com.*

